# Supplementary material for: MetaRibo-Seq measures translation in microbiomes
Source: Nat Commun. 2020 Jun 29;11:3268. doi: 10.1038/s41467-020-17081-z (PMC7324362; doi:10.1038/s41467-020-17081-z)
Supplement: Supplementary file 10 — Supplementary Data 7 [file 41467_2020_17081_MOESM10_ESM.zip › File2/Confidence_VeryHigh_Taxonomy/180558_out.krona.html]

Javascript must be enabled to view this page.

members
magnitude
magnitudeUnassigned
count
unassigned
taxon
rank

180558\_out

9


SRS011239\_contig\_number\_contig-100\_13381.13381SRS013476\_contig\_number\_15960
2

superkingdom
7
2

1239
7
phylum

2
species

SRS063040\_contig\_number\_contig-100\_2211.19304SRS140513\_contig\_number\_contig-100\_37.106613
1897035

class
4
186801

186802
4
order

541000

SRS014412\_contig\_number\_2484SRS048164\_contig\_number\_15064
family
2

216572
2
family

2
genus
459786

1897011

SRS012849\_contig\_number\_33423
species
1

species
1
1262910

SRS013951\_contig\_number\_17218


SRS104400\_contig\_number\_contig-100\_2447.293131
1263029
1
species
